# The pillars of land plants: new insights into stem development

**DOI:** 10.1016/j.pbi.2018.04.016

**Published:** 2018-10

**Authors:** Antonio Serrano-Mislata, Robert Sablowski

**Affiliations:** 1 Instituto de Biología Molecular y Celular de Plantas, CSIC-UPV, 46022 Valencia, Spain; 2 Department of Cell and Developmental Biology, John Innes Centre, Norwich Research Park, Norwich NR4 7UH, United Kingdom

## Abstract

In spite of its central importance in evolution, plant architecture and crop improvement, stem development remains poorly understood relative to other plant organs. Here, we summarise current knowledge of stem ontogenesis and its regulation, including insights from new image analysis and biophysical approaches. The stem initiates in the rib zone (RZ) of the shoot apical meristem, under transcriptional control by DELLA and BLH proteins. Links have emerged between these regulators and cell proliferation, patterning and oriented growth in the RZ. During subsequent internode elongation, cell wall properties and mechanics have been analysed in detail, revealing pectin modification as a prominent control point. Recent work has also highlighted signalling to coordinate growth of stem tissues with different mechanical properties.

## Highlights

• New image analysis revealed 3D growth patterns in the rib zone of the shoot meristem.
• Organ boundary genes emerged as an important control point in early stem growth.
• Stem elongation correlates with changes in pectin and its interaction with cellulose.
• Stem growth requires coordination across tissues with different mechanical properties.

**Current Opinion in Plant Biology** 2018, **45**:11–17

This review comes from a themed issue on **Cell signalling and gene regulation**

Edited by **Jorge Casal** and **Javier Palatnik**

For a complete overview see the Issue and the Editorial

Available online 12th May 2018

**https://doi.org/10.1016/j.pbi.2018.04.016**

1369-5266/© 2018 The Authors. Published by Elsevier Ltd. This is an open access article under the CC BY license (http://creativecommons.org/licenses/by/4.0/).

## Introduction

Development of a vertical shoot axis capable of bearing organs above the ground was one of the key steps in the evolution of land plants. The shoot of modern land plants is thought to derive from the sporophyte of a bryophyte-like ancestor, which acquired indeterminate growth maintained by apical initial cells [1, 2]. These apical initials were the precursors of the shoot apical meristem (SAM), and the first organ they produced was likely the equivalent of the stem in seed plants. Indeterminate growth and branching of the shoot axis set the stage for the evolution not only of overall shoot architecture, but also of leaves and probably roots [1, 2]. Therefore, development of the main axis of the shoot, represented in seed plants by the stem, is an ancient and fundamental aspect of plant development.

The control of stem elongation is also of great importance in crop improvement. Apart from the direct use of stems in the production of fibres, yarns, paper, wood and bio-fuels [3, 4], changes in stem height and architecture have been a key factor for increases in grain yields the last 50 years [5]. Semi-dwarf mutants played a key role in the ‘green revolution’ that averted a food security crisis in the 1960–70s and continue to be important in commercial varieties. The yield gain associated with shorter stems is attributed to an increase in harvest index (yield relative to total crop biomass) and to decreased lodging (falling over) caused by wind and rain [6]. Not all effects of these mutations, however, are advantageous for crop productivity. The regulatory genes affected control a large number of genes and processes and consequently their mutations have pleiotropic, undesired effects, for example, on seed size or seedling establishment [7, 8]. More precise genetic tools for modifying stem growth and architecture are needed to increase crop yield.

In spite of its basic and strategic importance, stem development has been relatively neglected, partly because ontogenesis of the stem is not as easily imaged as that of roots and lateral organ primordia, and perhaps because, at least externally, stems lack the attractive morphological complexity of leaves and flowers. Here, we review recent insights on stem ontogenesis and its regulation. We focus primarily on the initiation of stem tissues and on internode elongation, as secondary growth of the stem has been recently reviewed [9, 10].

## Initial stages of stem development: rib zone function

**Figure 1:**
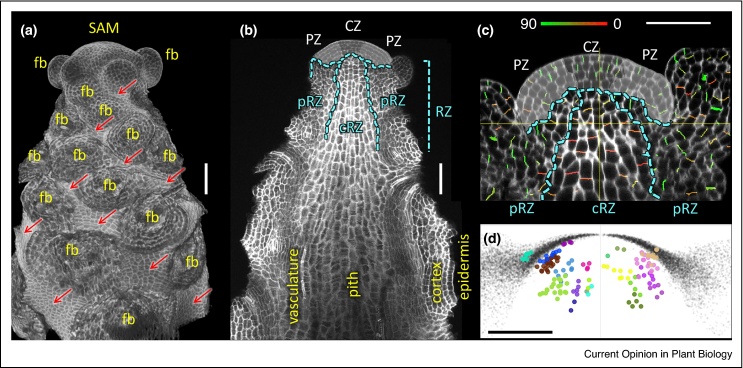
Early stages of inflorescence stem growth. **(a)** Three-dimensional (3D) view based on a stack of confocal images, showing the Arabidopsis inflorescence apex with most floral buds removed; SAM: shoot apical meristem; fb: position of floral buds; red arrows indicate the proliferating internode epidermis. **(b)** Longitudinal section through the image in (a), with the central zone (CZ), peripheral zone (PZ) and rib zone (RZ) indicated; the RZ is subdivided into central (cRZ) and peripheral (pRZ) regions; different stem tissues are indicated in yellow. **(c)** Close up of a SAM section similar to (b), with different SAM regions indicated as in (b); planes of recent cell divisions are coloured according to their orientation in 3D (the colour bar above the image shows angles between the stem main axis and the vector normal to a plane fitted to the new cell wall; red indicates divisions perpendicular to the main axis, green marks divisions at a low angle to the main axis). **(d)** Clonal analysis showing growth patterns in the SAM; each set of coloured dots indicates the position of cells descended from a single cell that had been genetically marked three days before imaging; clones were imaged in different apices, which were aligned and overlapped based on the position of floral buds; grey dots correspond to the position of epidermal cells in each of the overlapped apices. Bars: 50 μm. For details of methods used in (c) and (d), see [14^••^].

Like all other post-embryonic shoot organs, the stem is initiated at the SAM. Lateral organs, such as leaves and floral buds, are initiated in the SAM peripheral zone (PZ), which is replenished by the descendants of stem cells present in the central zone (CZ) [11]. In seed plants, the stem originates from the sub-apical region of the SAM, named the rib zone (RZ) because of its characteristic pattern of oriented cell divisions (Figure 1). The RZ includes a central region, which produces the pith of the stem, and a peripheral region that is continuous with the overlying PZ and gives rise to the stem epidermis and cortex; the stem vasculature develops at the boundary between both regions [12]. The lower boundary of the RZ is not anatomically clear, but the zone of active cell division typically extends to at least 1–2 cm from the apex [12].

In monocotyledons, stem elongation is also promoted by intercalary meristems (IMs) located at the base of each internode. Although located differently, the IMs are functionally comparable to the RZ in dicots and are subject to similar regulation. For example, both the RZ and IMs are the main sites where stem growth is stimulated by gibberellin (GA) [12, 13] and both are regulated by closely related BEL1-like (BLH) transcription factors, represented in Arabidopsis by REPLUMLESS (RPL) (also known as BELLRINGER, PENNYWISE, LARSON, VAAMANA and BLH9) and in maize by BLH12 and BLH14 [14••, 15•].

Early work showed that cell division in the RZ is stimulated by GA [12, 16]. This hormone acts by promoting the degradation of DELLA proteins [17], which modulate the activity of a wide set of proteins to integrate environmental and endogenous signals in plant growth. Recent work in both dicotyledons and grasses revealed the links between GA function and other hormones that regulate stem growth, particularly brassinosteroid (BR) and abscisic acid (ABA) [18, 19, 20, 21]. DELLAs were known to control cell division in the stem by binding and inhibiting the activity of class I TCP (TEOSINTE BRANCHED 1, CYCLOIDEA, and PROLIFERATING CELL FACTOR) transcription factors, which activate genes controlling cell cycle progression [22]. More recently, a direct link emerged between DELLAs and cell proliferation in the RZ, through activation of the cell-cycle inhibitor KRP2 (CDK/Kip-Related Protein2). Surprisingly, this revealed that control of cell division by DELLAs in the RZ affects not only stem elongation, but also the size of the overlying SAM and consequently the rate of floral bud production [23^•^].

KNOX-family transcription factors are central regulators of shoot meristem function in combination with BLH proteins [24]. Some of these, such as RPL in Arabidopsis, are particularly important for RZ development. RPL has been found to maintain RZ function by repressing the expression of organ boundary genes; in *rpl* mutants, ectopic expression of the *LSH4* (*LIGHT-DEPENDENT SHORT HYPOCOTYL 4*) boundary gene disrupted the distinct patterns of oriented cell division that distinguish the central and peripheral regions of the RZ (Figure 1) and inhibited stem elongation [14^••^]. Recent work highlighting the importance of oriented divisions in plant development suggests that they could be important for RZ function [25^•^]; alternatively, changes in oriented divisions and inhibited stem growth could be both consequences of changes in tissue patterning and hormone signalling downstream of *RPL* and *LSH4* [14^••^].

In addition to *LSH4*, RPL directly regulates numerous other organ boundary genes, several of which mediate the flowering and growth defects seen in *rpl* mutants [14••, 26, 27]. A subset of these, including *LSH4*, *LOB* (*LATERAL ORGAN BOUNDARIES*) and *CUC* (*CUP-SHAPED COTYLEDONS*) genes, are also directly targeted by DELLA proteins [23^•^], suggesting that organ boundary genes are particularly important in the control of stem elongation. One reason for this could be the known role of organ boundary genes in antagonising hormone signals, exemplified by the role of *LOB* in activating BR catabolism [28, 29]. Thus, boundary genes could regulate stem growth by restricting signals that control stem elongation, such as BR, which regulates GA synthesis in rice [21] and Arabidopsis [18] and whose role in stem elongation has been highlighted by dwarf mutants affected in BR synthesis in diverse plants, including barley [30], maize [20] and apple trees [31].

A prominent aspect of the early stages of stem development is the establishment of the vascular pattern. As in the rest of the plant, the path of new stem veins is established by self-reinforcing auxin flow from new organ primordia towards pre-existing veins [32], resulting in a vascular network that tracks the phyllotactic pattern established in the SAM [33]. In dicotyledons, this vascular network forms at the boundary between the peripheral and central regions of the RZ, possibly because, as in the SAM, auxin signalling is inhibited in the central region [34]. Therefore, the role of RPL in maintaining the radial pattern of the RZ, as described above, may contribute to defects in the vascular pattern seen in *rpl* mutants [35, 36]. However, RPL must also regulate vascular patterning through other mechanisms, because close homologs of RPL control the formation of vascular connections in the maize stem, where vascular bundles are dispersed instead of arranged around a central stem pith [15^•^].

## Later stages of stem development: internode elongation

**Figure 2:**
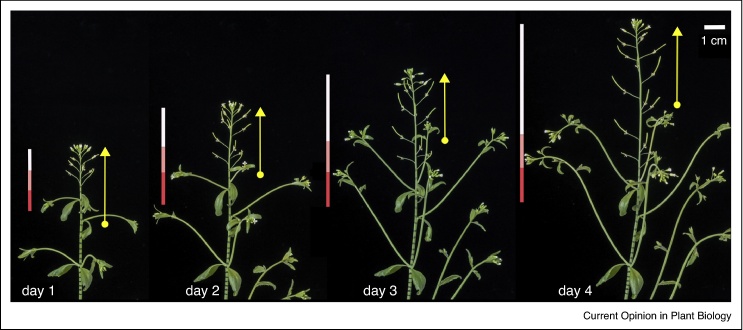
Internode elongation in Arabidopsis. The images show the same Arabidopsis inflorescence photographed at 1-day intervals; to facilitate growth tracking, inks marks were placed on part of the stem on day 1; yellow arrows on the right show the regions that continued to grow in subsequent time points; the bars on the left of each image track growth of different regions of the stem in day 1 (0–1 cm: white; 1–2 cm: pink; 2–3 cm: red). Note that the growth rapidly ceases below the proliferative region (white), which is responsible for most of the stem elongation seen in the following days.

As for other plant organs, the initial internode growth based on cell proliferation is gradually replaced by growth based on cell expansion, after which cells complete their differentiation. The relation between cell proliferation and expansion in internode elongation has been measured in detail in the Arabidopsis inflorescence stem: relatively slow growth by cell proliferation near the apex is followed by rapid but brief cell expansion, which ceases a few centimetres from the meristem after a three-fold increase in cell length [37]. The slower but sustained proliferative growth near the apex stem is responsible for most of the increase in stem length over a period of several days [14^••^] (Figure 2). Differential growth during the period of rapid cell expansion, however, is particularly important to adjust stem growth to changeable conditions, as seen in tropisms [38], propriosensing [39^••^] and in circadian tracking of the sun by sunflower heads [40^••^].

Regardless whether tissues grow predominantly through cell proliferation or cell expansion, in both cases growth is limited by the mechanical properties of cell walls. Earlier work tracked changes in cell wall composition and in the expression of cell wall-related genes during stem elongation [41, 42, 43] and recent papers showed that regulators such as RPL and DELLA proteins directly interact with genes involved in cell wall function [14••, 23•, 36, 44]. A detailed analysis of cell wall polysaccharide dynamics and mechanical properties in the apical region of the Arabidopsis inflorescence stem showed little change in cellulose composition, cell wall thickness or turgor pressure, and suggested that changes in the association between pectin and cellulose microfibers have a predominant role in the control of internode elongation [45^••^]. The prominent role of pectin changes during internode growth is in line with earlier evidence that RPL promotes internode elongation by activating expression of the pectin methylesterase PME5 [46].

The studies above revealed correlations between growth and the average properties of cell walls. However, stems are made of heterogeneous cell types, with different geometry and cell wall properties, raising the question whether specific tissues are rate-limiting for growth. Considering that the epidermis imposes a mechanical restraint on plant growth [47], the SAM can be mechanically modelled as an inflated shell [48]. If, similarly, the growing stem behaved as a cylinder under pressure, epidermal cells would be under tension in transverse orientation. Tissue contraction after cutting and the orientation of cortical microtubules, however, showed that the stem epidermis is under tension along the main axis of the stem, suggesting that although the epidermis may limit growth rate, inner tissues determine the direction of axial growth [49].

Thus, maintaining stem structure requires coordinated growth of tissues with different mechanical properties. This has been strikingly illustrated in double mutants for *CLAVATA3* and *DE-ETIOLATED 3 (clv3-8 det3-1)*, in which pressure exerted by excess cells in the central tissues, combined with inability to accommodate extra growth by cell expansion in the outer tissues, leads to spontaneous rupture of the inflorescence stem [50]. Mechanical stress that builds up during growth must be sensed by cells to adjust their wall properties. The highly conserved DEK1 Ca++-dependent calpain has been implicated as a component of this mechanosensing mechanism [51^••^]. Reflecting the importance of DEK1 in regulating cell wall properties, *DEK1* knockdown reduced deposition of cellulose and pectin, resulting in weak stems [52].

Coordination between tissue layers is mediated not only through tissue mechanics, but also chemical signalling, as demonstrated for epidermis-produced BR [53] and very long chain fatty acids (VLCFA) [54]. Reinforcing the importance of epidermal signals in shoot growth, it has been shown that during hypocotyl elongation auxin has specific roles in the epidermis, including the control of BR synthesis and signalling [55^•^]. It remains to be seen whether this also applies to the more complex growth of the post-embryonic stem. Conversely, internal tissues also produce signals that coordinate stem growth; for example, the LRR receptor ERECTA (ER) is required specifically in the phloem, where it receives peptide signals produced in the stem endodermis to regulate cell numbers across stem tissues [56, 57].

Cell differentiation eventually leads to the cessation of growth, associated with formation of secondary walls. Lignified secondary walls in the vascular bundles have a predominant effect on the mechanical properties of mature stems [3], therefore, the timing of vascular differentiation could also be expected to have a role in the control of internode elongation. However, a careful analysis of cell wall changes showed that lignin levels were constant throughout the elongating region of the Arabidopsis inflorescence stem [45^••^], suggesting that lignification mostly contributes to changes in stem mechanics during secondary growth, after elongation has ceased.

## Conclusions

Classic studies in the 1950–60s analysed in detail morphological and physiological aspects of early stem development. Since then, stem elongation has received relatively little attention, in contrast to stem secondary growth and vascular development. However, recent technical advances in quantitative imaging [14••, 23•] and biophysical techniques [3, 45••, 48] have allowed to improve our understanding of the cellular and mechanical bases of stem growth, and how these are influenced by regulatory genes. Stem development is not only a neglected but economically important process, but now also a convenient system to address plant developmental questions on its own right.

Despite the recent progress, many essential questions remain unanswered. Clonal analysis suggests that ground tissues of the RZ descend from the same stem cell population that gives rise to lateral organs (Figure 1) [14^••^]. This raises the question about how stem initiation is controlled separately from the emergence of other organs from the SAM. The initial stages of vascular development in the RZ are also poorly understood. Much more work is needed to fully understand how the growth of different tissues is coordinated during stem elongation and to identify the non-cell autonomous signals that mediate this coordination. Future research lines should also explore how environmental signals are integrated in the shoot apex to modulate RZ activity and stem growth. State-of-the-art developmental genetics, bio-imaging and biophysical techniques applied to stem development will continue to advance our understanding of this central process in plant development and will reveal more precise ways to modify stem architecture for crop improvement.

## References and recommended reading

Papers of particular interest, published within the period of review, have been highlighted as:

• of special interest
•• of outstanding interest
